# Cysteine metabolic circuitries: druggable targets in cancer

**DOI:** 10.1038/s41416-020-01156-1

**Published:** 2020-11-23

**Authors:** Vasco D. B. Bonifácio, Sofia A. Pereira, Jacinta Serpa, João B. Vicente

**Affiliations:** 1 iBB—Institute for Bioengineering and Biosciences, Instituto Superior Técnico, Universidade de Lisboa, Avenida Rovisco Pais, 1049-001 Lisboa, Portugal; 2 CEDOC, Chronic Diseases Research Centre, NOVA Medical School Faculdade de Ciências Médicas, Universidade NOVA de Lisboa, Campo dos Mártires da Pátria, 130, 1169-056 Lisboa, Portugal; 3 Instituto Português de Oncologia de Lisboa Francisco Gentil (IPOLFG), Rua Prof Lima Basto, 1099-023 Lisboa, Portugal; 4 Instituto de Tecnologia Química e Biológica António Xavier (ITQB NOVA), Avenida da República (EAN), 2780-157 Oeiras, Portugal

**Keywords:** Cancer microenvironment, Cancer metabolism

## Abstract

To enable survival in adverse conditions, cancer cells undergo global metabolic adaptations. The amino acid cysteine actively contributes to cancer metabolic remodelling on three different levels: first, in its free form, in redox control, as a component of the antioxidant glutathione or its involvement in protein *s*-cysteinylation, a reversible post-translational modification; second, as a substrate for the production of hydrogen sulphide (H_2_S), which feeds the mitochondrial electron transfer chain and mediates per-sulphidation of ATPase and glycolytic enzymes, thereby stimulating cellular bioenergetics; and, finally, as a carbon source for epigenetic regulation, biomass production and energy production. This review will provide a systematic portrayal of the role of cysteine in cancer biology as a source of carbon and sulphur atoms, the pivotal role of cysteine in different metabolic pathways and the importance of H_2_S as an energetic substrate and signalling molecule. The different pools of cysteine in the cell and within the body, and their putative use as prognostic cancer markers will be also addressed. Finally, we will discuss the pharmacological means and potential of targeting cysteine metabolism for the treatment of cancer.

## Background

**Fig. 1:**
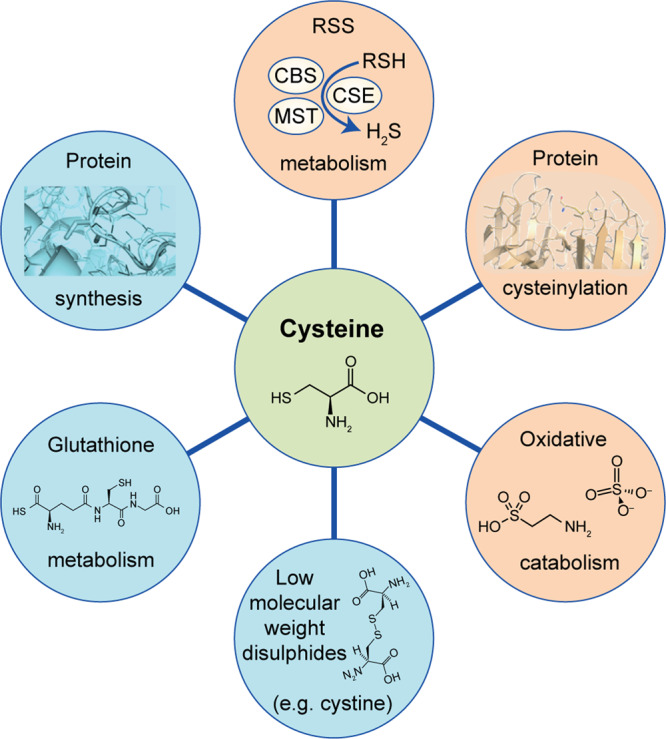
Cysteine metabolic fate. Cysteine has different fates, including the synthesis of glutathione or proteins, oxidative or non-oxidative catabolism and reversible post-translational protein modification (protein cysteinylation, production of reactive sulphide species and oxidation to cysteine disulphides).

Cysteine is a sulphur-containing proteinogenic amino acid; it has a free thiol group, which is likely to confer particular properties on functional sites of proteins that contain this highly conserved residue. As a multifaceted precursor, cysteine contributes to the survival and proliferation of cancer cells. Besides being a component of proteins and glutathione, cysteine is an important source of energy and biomass (Fig. 1).

**Fig. 2:**
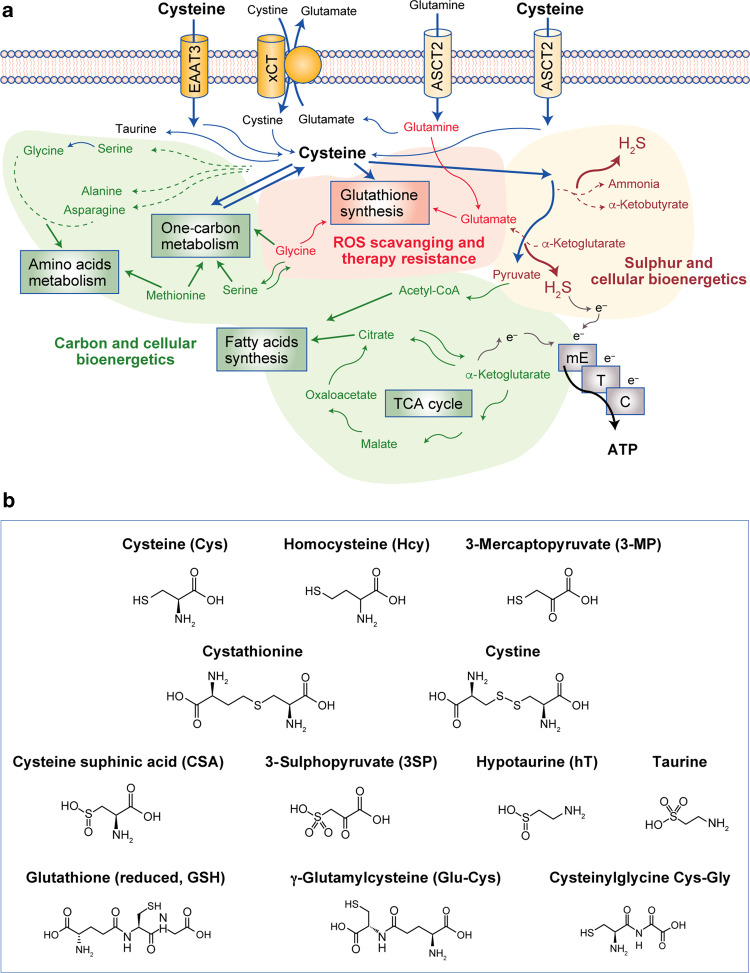
Cysteine: an intermediate and a supplier of several metabolic pathways. **a** Cysteine can be taken up in the form of cystine, through the cystine–glutamate antiporter transport system (xCT), or as cysteine through the excitatory amino acid transporter 3 (EAAT3) or the alanine-serine-cysteine-transporter 2 (ASCT2). Cysteine metabolism is tightly linked to that of glutamine, forming a network of amino acids capable of supplying the core metabolic pathways that underlie pivotal processes in cancer: reactive oxygen species (ROS) scavenging and chemoresistance dependent on glutathione synthesis; carbon and energy metabolism through fatty acid synthesis and the tricarboxylic acid (TCA) cycle, one-carbon metabolism and the production of ATP by the mitochondrial electron transfer chain (mETC), and sulphur and energy production as a generator of hydrogen sulphide (H_2_S), an electron (e^−^) donor for the mETC. **b** Cysteine (Cys) is a precursor of other organic compounds, such as homocysteine (Hcy), 3-mercaptopyruvate (3-MP), cystathionine, cystine, cysteine sulphinic acids (CSA), 3-sulphopyruvate (3-SP), hypotaurine (hT), taurine, glutathione (reduced, GSH), γ-glutamyl-cysteine (Glu-Cys) and cysteinylglycine (Cys-Gly).

Cancer cells face a range of intrinsic and extrinsic adverse/stressful conditions, such as nutrient and oxygen deficiency, and have consequently developed means to adapt their metabolism in order to survive. Cysteine has three main roles in the metabolic rewiring of cancer cells: as a precursor of glutathione under the action of glutamyl-cysteine ligase (GCL), contributing to oxidative stress control; as a substrate for the production of hydrogen sulphide (H_2_S), which stimulates cellular bioenergetics; and as a carbon source for biomass and energy production (Fig. 2a). Cysteine is also essential for the ability of cancer cells to evade drug exposure and cell injury and adapt to other stressful conditions such as hypoxia. As cysteine and glutathione are scavengers of free radicals (mainly reactive oxygen species (ROS)), they can abrogate the effects of the majority of oxidative or alkylating drugs used in cancer therapy, affording an important resistance mechanism.^1–7^ Glutathione is also a highly important component in detoxification, allowing the physiological and pathophysiological maintenance of cell metabolism.^8–11^ Moreover, the relevance of cysteine in the production of other organic compounds and H_2_S (Fig. 2a, b) highlights the importance of the bioavailability of cysteine to enable cells to adapt to metabolically challenging conditions, as well as mediating cancer cell survival, tumour growth, metastases formation, resistance to therapy and disease recurrence.

In this article, the cellular and systemic roles of cysteine in the metabolic remodelling that occurs in cancer cells will be outlined. Emphasis will be placed on the metabolic pathways and relevant players (Table 1) that interfere with cysteine anabolism and catabolism, and the determinants that affect cysteine bioavailability, thereby influencing cancer development. Finally, insights into the pharmacological targeting of cysteine metabolism will be presented.

**Table 1:** Cysteine metabolism-related players in cancer.

Enzymes	Cancer-related alterations	Refs.
Cysteine dioxygenase (CDO)		
	↑ CDO:	
	- Good prognosis	^70^
	- Bad prognosis	^76^
	- ROS production	^71^
	- Toxicity	^70^
	- ↓ OXPHOS	^76^
	- Ferroptosis inhibition	^75^
	↓ CDO:	
	- Poor prognosis	^71,74^
	- Drug resistance	^71^
Cystathionine β-synthase (CBS)		
	↑ CBS:	
	- Cancer malignancy	^58,60,56^^-^^61^
	- ↓ ROS production	^58,109,153^
	- ↑ OXPHOS	^58,109,153^
	- ↑ Proliferation	^170^
	- ↑ Migration	^170^
	- ↑ Angiogenesis	^156^^-^^159^
	- Ferroptosis inhibition	^85,171^
Cystathionine γ-lyase (CSE)		
	↑ CSE:	
	- Cancer malignancy	^54,55,57,59,61^
	- ↑ Angiogenesis	^156^^-^^159^
	- Apoptosis inhibition	^168^
3-Mercaptopyruvate sulphurtransferase (3-MST)		
	↑ 3-MST:	
	- Cancer malignancy	^57,59,61^
	- ↑ OXPHOS	^114,147,148^
Cysteine desulphurase (NFS1)		
	↑ NFS1:	
	- Ferroptosis inhibition	^138,140^
	↓ NFS1:	
	- Chemoresistance	^138^
Sulphide:quinone oxidoreductase (SQR)		
	↑ SQR:	
	- Hypoxia related	^154^
	↓ SQR:	
	- ↓ OXPHOS	^114,148^
Transporters	Cancer-related alterations	Refs.
Alanine-serine-cysteine transporters 1 (ASCT1; *SLC1A4*)		
	↑ ASCT1:	
	- Cancer malignancy	^35,38,39,41^^-^^43^
	- ↑ Glutamine metabolism	^41^^-^^43^
Alanine-serine-cysteine transporters 2 (ASCT2; *SLC1A5*)		
	↑ ASCT2:	
	- Cancer malignancy	^35,38,39,41^^-^^43^
	- ↑ Glutamine metabolism	^41^^-^^43^
Excitatory amino acid transporter 3 (EAAT3; *SLC1A1*)		
	↑ EAAT3:	
	- Cancer malignancy	^35,38,39^
	- Chemoresistance	^38^
Neutral and basic amino acid transporter (RBAT; *SLC3A1*)		
	↑ RBAT:	
	- ↓ ROS	^33^
	- ↑ Cancer cell survival	^33^
xc^−^ transport system (xCT; *SLC7A11*)		
	↑ xCT:	
	- Cancer malignancy	^25^^-^^27,180^
	- Chemoresistance	^7,236^
	- ↓ ROS	^7^
	- Ferroptosis inhibition	^217,218,221^
	↓ xCT:	
	- TSP activation	^29,32^

Presentation of the association between the expression and activity of enzymes and transporters and cancer metabolic alterations and disease progression.

## Cysteine bioavailability: main players and regulators

As mentioned above, the bioavailability of cysteine in a cancer cell can influence metabolic fitness and the development of therapy resistance. Although cysteine can be derived from the catabolism of extracellular glutathione, protein catabolism or de novo synthesis from methionine,^12–14^ the major source of cellular cysteine is the dietary intake of cystine, the oxidised form of cysteine.^12,15^ The oxidative environment of the plasma favours free cysteine dimerisation into cystine, which then becomes the predominantly available form for cells to take up from the surrounding milieu.

### Cystine transporters

A number of cystine transporters have been described and studied in the context of cancer; however, as some of these transporters are cystine–glutamate antiporters (Fig. 2),^16,17^ many of these studies have focused on the relevance of glutamate, mainly in the central nervous system, describing a correlation between increased glutamate efflux and increased cancer aggressiveness and invasive capacity (and, consequently, poor prognosis).^16,18–22^ As cystine influx through the cystine–glutamate xCT antiporter occurs concomitantly with glutamate efflux, increased cystine import can also be related to poor prognosis. Thus, cancer cells in metabolic and phenotypic equilibrium can be disturbed by blocking cyst(e)ine/glutamate transporters.^16,23,24^

Moreover, in cancer cell lines (Table 1), increased expression levels of xCT were found to be associated with increased intracellular levels of glutathione and cisplatin resistance.^7^ In fact, xCT is considered to be the main transporter of cystine in cancer (Table 1)^25–27^ and its expression is controlled by nuclear factor erythroid 2-related factor 2 (NRF2), a master regulator of the cellular redox state,^28^ highlighting the importance of xCT in an oxidative stress-resistant cancer cell phenotype. In addition, xCT expression can be regulated by the phosphoinositide 3-kinase/protein kinase B/mammalian target of rapamycin (PI3K/PKB/mTOR)^29–31^ and mitogen-activated protein kinase (MAPK) pathways in synergy with activating transcription factor 4 (ATF4), which is activated by endoplasmic reticulum stress.^27^ Decreased expression of xCT leads to an increase in the rate of sulphur transfer from homocysteine to cysteine (through the trans-sulphuration branch of the methionine cycle), supporting the pivotal role of xCT in cyst(e)ine transport and cysteine provision.^29,32^ The neutral and basic amino acid transporter (RBAT, also designated by *SLC3A1*) is another cystine transporter with possible implications in cancer development. Its expression has been linked to the capacity of breast cancer cells to control their redox state by promoting the accumulation of reduced glutathione, thereby decreasing ROS levels and increasing cell survival (Table 1).^33^

### Non-specific cysteine transporters

While influx transporters are the main means by which cells acquire cystine, cysteine can also be taken up from the extracellular milieu into cells directly by excitatory amino acid transporter 3 (EAAT3) and the alanine-serine cysteine transporters 1 and 2 (ASCT1/2), all of which are known to be overexpressed in different cancer types^34^ (Table 1). However, the association between cysteine transport and the overexpression of these transporters in cancer metabolic remodelling has not yet been established, as these transporters are not specific for cysteine and so the focus of most studies is the transport of other amino acids (e.g. glutamine and glutamate).

A limited number of studies have investigated EAAT3 in cancer. For example, the role of EAAT3 was explored in brain tumour models, but the studies focused on glutamate transport and the central nervous system-specific glutamatergic cycle,^35,36^ which is essential for the ultimate production of neurotransmitters.^37^ Nonetheless, EAAT3 has been associated with increased chemoresistance in colorectal cancer models^38^ and reported to be highly expressed in prostate cancer.^39^ ASCT1 and ASCT2, which have mainly been studied in the context of glutamine dependence, are expressed at high levels in different cancer types,^40–42^ prompting these transporters to be considered putative therapeutic targets in cancer, with different inhibitors currently under investigation.^40,43–45^ Furthermore, glutamine and cysteine metabolism are deeply linked,^46,47^ because serine and glycine can derive from glutamine and, by entering the one-carbon metabolism pathway, they will contribute to homocysteine and cysteine syntheses.^1^ This fact makes ASCT1, ASCT2 and EAAT3 pivotal in the reliance of cancer cells on cysteine uptake and anabolism.

## Cysteine catabolism and its interplay with other metabolic pathways in cancer

Cysteine plays a central role in cellular metabolism as a key component of carbon and sulphur metabolism (Fig. 2). There are two main pathways for cysteine catabolism: one is via its enzymatic breakdown to produce H_2_S and organic intermediates that will serve as carbon sources, and the second is via oxidative metabolism through cysteine dioxygenase (CDO). The production of H_2_S and its role in bioenergetics and signalling will be addressed later in the article.

### Cysteine degradation and energy metabolism

**Fig. 3:**
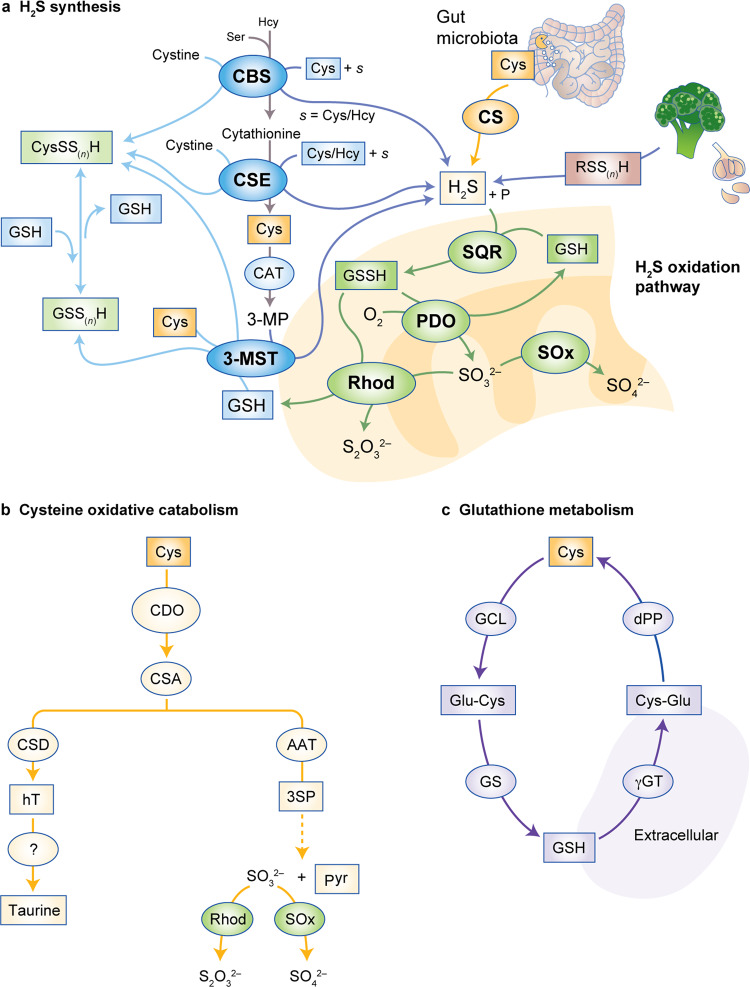
Metabolic pathways involved in cysteine catabolism. Cysteine can be a substrate for hydrogen sulphide (H_2_S) synthesis, be oxidatively catabolised to taurine or be a substrate for glutathione production. **a** The trans-sulphuration pathway. The enzymes cystathionine β-synthase (CBS) and cystathionine γ-lyase (CSE) catalyse the conversion of homocysteine into cysteine, generate hydrogen sulphide through several alternative reactions or cysteine per/polysulphide (CysSS_(*n*)_H) with cystine as substrate. Cysteine is converted by cysteine aminotransferase (CAT) into 3-mercaptopyruvate (3-MP), which is a substrate of 3-MP sulphurtransferase (3-MST), of which there is a cytosolic and a mitochondrial isoform. 3-MST can also generate CysS_(*n*)_SH and glutathione per-/polysulphide (GS_(*n*)_SH), respectively, using Cys and GSH as sulphur acceptors. H_2_S is catabolised by a mitochondrial sulphide oxidation pathway. H_2_S is a substrate of sulphide:quinone oxidoreductase (SQR), which preferentially uses glutathione as a sulphur acceptor, to generate oxidised glutathione (GSSH) that is converted back into GSH by persulphide dioxygenase (PDO) using oxygen as co-substrate, yielding sulphite (SO_3_^2−^) as co-product. Oxidised glutathione is also a substrate of rhodanese (Rhod), which uses SO_3_^2−^ as co-substrate to yield thiosulphate (S_2_O_3_^2−^) and glutathione. SO_3_^2−^ oxidation by sulphite oxidase yields sulphate (SO_4_^2−^). **b** Cysteine oxidation. Cysteine can be oxidised by cysteine dioxygenase (CDO) to cysteinesulphinate (CSA), which is converted by cysteinesulphinate decarboxylase (CSD) into hypotaurine (hT), which is further oxidised to taurine. Alternatively, cysteinesulphinate can be transaminated by aspartate aminotransferase (AAT) to 3-sulphinopyruvate (3-SP), which decomposes to form pyruvate and sulphite/sulphate. **c** Cysteine is a substrate for glutathione generation. Cysteine is converted into γ-glutamyl-cysteine (Glu-Cys) by glutamate-cysteine ligase (GCL) and subsequently to glutathione by glutathione synthase (GS). Conversely, GSH is converted into cysteinylglycine (Cys-Gly) by γ-glutamyl transpeptidase (γGT) and finally back to cysteine by dipeptidase (dPP).

Although the role of cysteine degradation in cancer development has predominantly been explored regarding H_2_S production, the usefulness of cysteine as a carbon source is also evident along its catabolic pathways, as its degradation gives rise to other organic compounds relevant for carbon and energy metabolism (Fig. 2). These compounds include pyruvate, which can be converted into acetyl-CoA and enter the tricarboxylic acid (TCA) cycle or be used for fatty acid synthesis, and α-ketoglutarate, a precursor of glutamate and an intermediate of the TCA cycle.^48–51^ Moreover, through the action of two of the enzymes involved in the production of H_2_S—cysteine aminotransferase (CAT) and 3-mercaptopyruvate sulphurtransferase (3-MST)—cysteine is sequentially converted into 3-mercaptopyruvate (3-MP) with the release of an amino group that will react with α-ketoglutarate, ending with the formation of glutamate and pyruvate (Fig. 3a), again connecting cysteine metabolism with the TCA cycle. Given the increased expression of cysteine catabolic enzymes, such as cystathionine β-synthase (CBS), cystathionine γ-lyase (CSE) and 3-MST in different cancer cell types, it is likely that their relevance for cancer development is shared between the production of H_2_S (detailed later in the article) and the accompanying generation of metabolites that constitute carbon sources.^52–60^

### Cysteine-oxidative catabolism

In the second pathway for cysteine catabolism, CDO catalyses the conversion of cysteine into cysteine sulphinic acid (CSA; Figs. 2b and 3b), leading to the cellular production of taurine, pyruvate (Fig. 2b) and sulphate.^61–63^ CDO is highly regulated at the level of protein turnover, as oxidative degradation of cysteine by CDO is highly inducible,^64^ and high cysteine levels inhibit CDO ubiquitinylation and reduce its proteasomal degradation.^65^ Deficient CDO activity has been related to lower sulphate levels in plasma, elevated fasting plasma cysteine concentrations, and lower sulphate-conjugate:glucuronide-conjugate ratios for paracetamol detoxification products, consistent with impaired cysteine catabolism.^66^ CDO competes with GCL (Fig. 3c) for cysteine and thereby contributes to the regulation of the intracellular availability of cysteine and glutathione^67^ and to H_2_S production.^68^

In non-transformed cells, NRF2 promotes the entry of cysteine into the taurine synthesis pathway via CDO1,^69^ and modestly limits the accumulation of cysteine and glutathione synthesis. In cancer, CDO has been shown to divert the flow of cysteine away from glutathione synthesis, thereby promoting the production of ROS.^70^ As cells use NADPH to reduce cystine to cysteine, the increased cysteine catabolism by CDO implies the increased consumption of NADPH, which will affect other cell metabolic pathways and the antioxidant capacity as well. Cysteine starvation or xCT blockade confers the opposite result and increases cellular NADPH pools.^69^

The important role of taurine as a cellular osmoregulator will not be described in detail in this paper. However, it should be mentioned that taurine levels contribute to cellular homeostasis, and that this role is particularly important in the brain^71^ and kidney.^72^ The overexpression of components of cysteine catabolic pathways in cancer cells is likely to result in increased pyruvate production. This link between cysteine-oxidative catabolism-derived pyruvate and cancer has been described in pancreatic cancer^73^ and non-small lung cancer cells,^69^ although additional studies that focus on other cancer types are required to strengthen this association.

### CDO1: a tumour suppressor?

The predominant notion, supported by a number of reports in different cancer types (detailed below), is that CDO1 is a tumour suppressor gene simply by virtue of its silencing in cancer; however, it is ought to be confirmed. In cancer (Table 1), CDO1 is usually silenced by promoter methylation and this is associated with poor prognosis,^70,74^ implying that the shutting down of CDO1 expression favours cancer progression. The CDO products sulphite and CSA are toxic to lung cancer cells. As cysteine stabilises CDO levels, cells that express high levels of NRF2 are sensitive to CDO-related toxicity.^69^ Taurine is also a marker of CDO activation, and lower levels of taurine in lung cancer cells are related to higher intracellular cysteine concentrations.^69^ In gastric cancer cells, the absence of CDO1 contributed to restore cellular glutathione levels, to prevent ROS and lipid peroxidation, and to promote resistance to ferroptosis, a type of programmed cell death dependent on iron and associated with lipid peroxide accumulation.^75^ Conversely, forced overexpression of CDO1 in breast cancer cells shifted the flux from glutathione synthesis towards cysteine catabolism, consequently increasing ROS levels and leading to reduced cell viability and growth.^70^ In breast cancer, hypermethylation of the CDO1 promoter, which is frequently observed, is a predictor of poor outcome in anthracycline-treated, oestrogen receptor-positive and lymph-node-positive patients.^70^ CDO1 expression might therefore be a useful indicator for the prediction of drug resistance, and hypomethylating agents (e.g. 5-azacytidine) might have a role in the treatment of breast cancer cells with epigenetically silenced CDO1—for example, by inducing sensitisation to anthracycline. As anthracyclines are ROS-generating drugs, one of the resistance mechanisms might be related to the ability of cancer cells to evade this insult.

However, in contrast to the tumour-suppressive effects described above, there is evidence that CDO1 can have a tumorigenic effect associated with aggressive glioblastoma, since its overexpression was detected in these tumours.^70,74^ Patient-derived glioblastoma specimens exhibit overexpression of CDO1 and consequent accumulation of CSA.^76^ Altogether, although many data point towards CDO1 as being a tumour suppressor gene and a putative prognostic marker for cancer outcome, divergent evidence from glioblastoma patient samples highlight the need for more studies to disclose the actual impact of cysteine-derived organic compounds in cancer metabolic rewiring.

## Cysteine anabolism and its interplay with other metabolic pathways in cancer

### De novo cysteine synthesis

The de novo synthesis of cysteine occurs through the trans-sulphuration pathway of the methionine cycle, which also involves serine (Fig. 3a), and renders the synthesis of cysteine dependent on the activity of one-carbon metabolism (methionine and folate cycles).^77–79^ Dietary methionine is converted into homocysteine in two consecutive steps: the first involves the condensation of homocysteine with serine, catalysed by CBS to yield cystathionine (Fig. 2b), which is subsequently hydrolysed by CSE in the second step to generate cysteine and other compounds (e.g. ammonia, α-ketoglutarate or propionate), thus establishing a link between the trans-sulphuration pathway and the TCA cycle (as reviewed in Combs and DeNicola.^80^) Cancer cells express higher levels of the trans-sulphuration pathway enzymes CBS and CSE than normal cells (reviewed e.g. in refs.,^81,82^) highlighting this pathway as a crucial metabolic pathway in cancer cells.^83^ A very recent study presents the trans-sulphuration pathway as being directly beneficial for cancer cells to maintain the redox equilibrium and evade ferroptosis, as, upon inhibition of cysteine import, glutathione synthesis is supplied by cysteine derived from the trans-sulphuration pathway.^84^ In the same study, the concept was proven in cancer cell lines by reverting lipid oxidation through the addition of homocysteine. The relevance of the trans-sulphuration pathway in the survival of cancer cells and reversion of ferroptosis was also proven by silencing CBS, thus inhibiting cysteine synthesis.^85^

### One-carbon metabolism and cysteine bioavailability

The de novo synthesis of cysteine cannot be addressed without mentioning the involvement of serine and glycine in the one-carbon pathway. The serine synthesis pathway is mainly supplied by glucose-derived 3-phosphoglycerate, which is converted into 3-phosphohydroxypyruvate by the action of phosphoglycerate dehydrogenase, which also converts NADH into NAD^+^.^86^ Subsequently, 3-phosphohydroxypyruvate is converted into serine by two sequential reactions catalysed by phosphoserine aminotransferase 1 and phosphoserine phosphatase. Then, serine hydroxymethyltransferase converts serine into glycine, which will enter one-carbon metabolism.^78^ In cancer, c-Myc, a pivotal and well-known oncogene, is the main regulator of the serine synthesis pathway.^79,87^

Activation of the serine synthesis pathway correlates directly with glutathione synthesis,^87^ as serine-derived glycine is one of the components of glutathione. Serine and glycine are needed for cysteine synthesis in one-carbon metabolism, and serine-derived glycine is preferentially used by cancer cells over exogenous glycine.^88^ Therefore, in the context of cancer metabolic remodelling, the import of serine is a key control point of the one-carbon pathway.

One-carbon metabolism also plays a role in the regulation of gene expression and epigenetic modulation. Several types of cancer have their carcinogenesis associated with the silencing of physiologically beneficial genes, such as tumour suppressor genes, through DNA methylation,^89,90^ as described earlier in the article for CDO1. Furthermore, in some tumours, the need for methyl groups for epigenetic regulation prevents activation of the trans-sulphuration pathway for cysteine synthesis, instead prioritising one-carbon metabolism.^83^ The folate cycle uses glycine and tetrahydrofolate (converted from folic acid) and produces intermediates (5,10-methylene-tetrahydrofolate and 5-methylene-tetrahydrofolate) to supply methyl groups for purine synthesis. After the folate cycle, through the interconnection with the methionine cycle involving the entry of cobalamin (vitamin B12), folic acid is again synthesised.^91^ In the methionine cycle, methionine is sequentially converted into *s*-adenosyl-l-methionine (the methyl donor for all methylation reactions in the cell) and *s*-adenosyl-l-homocysteine.^92^ *s*-Adenosyl-l-homocysteine can be used for pyrimidine synthesis or converted into homocysteine, which can be re-methylated to methionine or enter the trans-sulphuration pathway to generate cysteine, through CBS and CSE.^50,93^ Cysteine catabolism depends on the methionine cycle, belonging to the one-carbon metabolism, which supplies different pathways relevant in carcinogenesis and cancer metabolism.

### Glutathione metabolism

Together with cysteine anabolism, the extracellular catabolism of glutathione constitutes a relevant source of cysteine.^12^ Following ROS scavenging, the degradation of oxidised glutathione through the γ-glutamyl cycle (Fig. 3c) facilitates recycling of cysteine, glycine and glutamate. Upon its efflux from cells, oxidised glutathione is exposed to the sequential action of enzymes located on the external face of the cell membrane: γ-glutamyl transpeptidase (GGT) generates glutamate^94^ and the cysteinylglycine dipeptide, which is degraded by dipeptidases such as aminopeptidase N (APN), thereby releasing cysteine and glycine.^95^ Glutamate, cysteine and glycine can then be imported by the cell using specific transporters. The cysteinylglycine dipeptide can also be taken up by cells in a process mediated by the peptide transporter 2, before being degraded in the cytoplasm by unspecific dipeptidases,^96^ although the circumstances under which this occurs remain undetermined.

Glutathione catabolism can also be carried out by CHAC1 and CHAC2 isoenzymes, which belong to the γ-glutamylcyclotransferase family.^97,98^ These enzymes catalyse the degradation of glutathione and the release of cysteinylglycine in the cytosol, rather than outside the cell as for GGT. Although not much is known about these enzymes in cancer, the CHAC2 isoform has already been classified as a tumour suppressor gene in gastric and colorectal cancer, on the basis of its downregulation being associated with more aggressive cancer variants and its activation-inducing apoptosis in cancer cells.^99^

## Cysteine-derived reactive sulphide species

Cysteine is a key player in cancer as a source of reactive sulphide species (RSS), particularly as a substrate for endogenous and gut-microbe-derived H_2_S-synthesising and -catabolising enzymes. Tight and intricate regulatory processes maintain physiological H_2_S levels, while imbalances either in its production or breakdown have deleterious consequences, with high H_2_S levels eventually becoming toxic and/or pathogenic. At low intracellular concentrations (0.01–1 μM), H_2_S injects electron equivalents into the mitochondrial electron transport chain (mETC) by reducing quinone to quinol via sulphide:quinone oxidoreductase (SQR), ultimately stimulating ATP production.^100^ However, at 3–30-fold higher concentrations, H_2_S becomes toxic essentially by inhibiting cytochrome *c* oxidase (KI 2.6 μM at mitochondrial pH 8.05; reviewed e.g. in refs.^100,101^). The implications of this effect of H_2_S on cellular bioenergetics in the context of cancer are discussed below.

The H_2_S-related RSS per-sulphides and poly-sulphides can be generated by the same enzymatic systems that are involved in H_2_S metabolism, and play a possibly as-yet-underestimated role in signalling in human health and disease,^102–104^ which is also briefly discussed below.

### Biosynthesis of H_2_S

H_2_S can be generated in mammalian physiology via dedicated endogenous enzymatic systems, gut microbiota metabolism and the breakdown of dietary polysulphide sources such as, for example, garlic and onion (reviewed e.g. in refs.^82,105–107^) (Fig. 3a). H_2_S is mainly generated by three endogenous enzymes with different organ/tissue distributions, all of which are related to cysteine metabolism: the trans-sulphuration pathway pyridoxal-5′-phosphate-dependent-CBS and CSE, and 3-MST.^105^ Historically considered to be cytosolic, CBS and CSE can also—under certain (patho)physiological conditions—translocate to mitochondria^108,109^ or nuclei,^110^ or even be secreted.^111^ 3-MST can be detected in mitochondria and in the cytosol.^112^ While H_2_S-mediated protein per-sulphidation is relevant in any cellular compartment, mitochondrial H2S generation is particularly relevant for cellular bioenergetics, as detailed later in the article. Secreted CBS and CSE have been proposed to contribute for the maintenance of homocysteine levels in the plasma.^111^ 3-MST uses 3-MP, derived from cysteine through the action of CAT, as an activating substrate to generate a persulphide at the catalytic Cys248 residue.^113^ H_2_S is then released upon the reaction of activated 3-MST with a sulphane sulphur acceptor such as thioredoxin, cysteine, homocysteine, glutathione, or even *n*-acetylcysteine, thus generating the corresponding per-sulphides.^114–116^ Whereas the canonical reactions catalysed by CBS and CSE within the trans-sulphuration pathway sequentially convert homocysteine into cysteine, both enzymes catalyse a number of alternative H_2_S-yielding reactions, using as substrates different combinations of these very same sulphur amino acids (reviewed e.g. in refs.^82,117^).

### Regulation of H_2_S and RSS levels

The reactivity of H_2_S and related RSS demands a tight control of their levels, which is ensured both by an intricate regulation of their synthesising enzymes and through an efficient sulphide detoxification pathway located in mitochondria. Whereas CSE and 3-MST are mostly regulated at the transcriptional level, CBS is functionally controlled by a number of post-translational modifications and interactions. The activity of CBS is increased by ~2–3-fold upon *s*-adenosyl-l-methionine binding to its C-terminal domain.^118^ Redox reactions at key cysteine residues—namely, *s*-glutathionylation at Cys346^119^ and reduction of the Cys272-X-X-Cys275 disulphide—also enhance CBS enzymatic activity.^119^ Conversely, at the N-terminal domain, a non-catalytic regulatory haem sensor negatively impacts the enzymatic activity of CBS upon reduction and completely inactivates the enzyme upon binding of nitric oxide or carbon monoxide.^100,120–122^ An N-terminal intrinsically disordered peptide sequence has been shown to bind another haem moiety, although its function remains to be determined.^123^ As CBS and CSE use the same substrates to generate H_2_S, their function is interdependently regulated through substrate/product accumulation/depletion, which will favour one biochemical pathway over the other. Indeed, inhibition of CBS can yield overall higher H_2_S production through CSE, which presents a higher catalytic efficiency.^124^

Sulphide levels are enzymatically controlled through the catabolic sulphide-oxidising pathway (SOP) located in mitochondria. The first (irreversible and committing) step is catalysed by SQR, which oxidises H_2_S and transfers the electron equivalents to coenzyme Q and the sulphur atom to an acceptor molecule that becomes persulphidated.^100,125,126^ The preferred sulphur-accepting substrate is still a matter of debate, but glutathione appears to be the most effective and plausible in physiological conditions.^127–130^ The resulting glutathione persulphide is a substrate for either persulphide dioxygenase, which oxidises the sulphane sulphur yielding sulphite and glutathione, or rhodanese, which generates thiosulphate. Finally, sulphite oxidase converts sulphite into sulphate.^125^ The expression and activity of the SOP enzymes appear to be strongly related to the H_2_S levels to which the corresponding cells or tissues are exposed. For example, whereas sulphide-oxidising activity is virtually undetectable in nervous system cells, colonocytes display high expression and activity of SOP enzymes,^131–133^ consistent with the high H_2_S concentrations that result from gut microbial metabolism. Indeed, the apical localisation of SOP enzymes in human colonic crypts places this pathway optimally at the host–microbiome interface.^134^

As well as H_2_S, several other RSS are synthesised in human physiology, and similarly fulfil a number of signalling functions, which result essentially from modification of, or interaction with, target proteins. H_2_S, for example, can bind to protein metal centres, such as haem moieties in mitochondrial cytochrome *c* oxidase or haemoglobin (reviewed e.g. in ref.^135^). Probably the most prevalent H_2_S-mediated modification of target proteins, however, involves per-sulphidation (or polysulphidation) of cysteine residues (i.e. the addition of sulphane sulphur) with the concomitant functional and/or structural consequences (reviewed e.g. in refs.^82,102,103^), some of which are described below in relation to cancer. The enzymatic pathways involved in H_2_S metabolism are per se sources of low molecular weight per-sulphides and poly-sulphides. Indeed, both CBS and CSE can synthesise cysteine persulphide (CysSSH) and poly-sulphides (CysS_(*n*)_SH) from cystine,^104,136^ whereas 3-MP-activated 3-MST can generate from cysteine, glutathione or *n*-acetylcysteine, the corresponding per-sulphides (respectively, CysSSH, GSSH and NACSSH).^114,115^ Within the sulphide oxidation pathway, GSSH is also generated mostly as a product of SQR. The mitochondrial cysteinyl-tRNA synthase (CARS2) has been identified as the main cellular source of CysSSH, yielding free CysSSH and co-translationally inserting persulphidated cysteine into nascent polypeptides.^137^ The intrinsic reactivity and peculiar chemistry of per-sulphides and poly-sulphides indicate that they might carry out numerous signalling functions that could currently be underestimated mainly owing to the technical difficulties in studying these metabolites in biological milieu.

### NFS1 and iron–sulphur clusters

Another enzyme capable of metabolising cysteine and producing a sulphur carrier is the mitochondrial cysteine desulphurase (NFS1). NFS1 degrades cysteine and releases sulphide, which can be used to generate iron–sulphur (Fe–S) clusters,^138^ versatile co-factors that carry out electron transfer, catalysis and afford structural stability. These Fe–S clusters are synthesised in the mitochondrion before being exported out by chaperones and channels to participate in the maturation of Fe–S proteins (reviewed in ref.^139^) The association between NFS1 activity and cancer relates to temporary abrupt increases in oxygen tensions experienced by cancer cells that differently affect tumours according to their tissue and organ localisation. Metastatic or primary lung tumours were shown to rely on enhanced expression of NFS1 to maintain the supply of Fe–S clusters as co-factors of multiple essential proteins and enzymes in the cell that are exposed to (damaging) high oxygen concentrations.^140^ Whereas the continuous supply of Fe–S clusters by NFS1 also prevented the iron-starvation response from being triggered in lung adenocarcinomas, NFS1 suppression was shown to predispose cancer cells to ferroptosis.^140^ Thus, cysteine contributes to the inhibition of ferroptosis both as a component of glutathione, which facilitates the scavenging of lipid peroxides by the phospholipid hydroperoxidase glutathione peroxidase 4 (GPX4), and as a substrate of NFS1. Accordingly, dysregulation of the Fe–S clusters biogenesis, including decreased expression of NFS1, has also been described as being relevant in mechanisms of resistance to cancer therapy.^138^ Therefore, the activation of ferroptosis seems to be an important goal in cancer therapy, and triggering NFS1 might be a suitable strategy.

## Cysteine-derived H_2_S in cancer development

A role for cysteine in cancer associated with disturbed metabolism and signalling of H_2_S, per-sulphides and poly-sulphides has been established on the basis of a range of evidence. In different cancer types, higher expression and activity, and changes in the localisation, of H_2_S-synthesising and -breakdown enzymes have been observed in cancer specimens or cell models (Table 1), as compared with tumour-adjacent normal tissue or non-tumorigenic cells, and associated with different aspects of cancer development (e.g. refs.^81,134,141^) In addition to perturbations in endogenous RSS metabolism, excessive H_2_S generated via bacterial cysteine desulphydrase from gut microbiota species such as *Fusobacterium nucleatum* has been linked to the development of colorectal cancer.^106,142^ The microbial influence in systemic and cellular metabolism is a new, controversial and developing field in cancer research. However, in colorectal cancer—the most explored cancer model in this matter—it seems that both microbially derived and endogenous H_2_S play a role in cancer progression and colon health.^106^

Following pioneering studies that linked CBS overexpression with colorectal and ovarian cancer (Table 1), an increase in the expression of all enzymes involved—individually or jointly—in the synthesis of H_2_S has also been reported for breast, gastric, lung, liver, bladder, kidney and prostate carcinomas, melanoma, neuroblastoma, glioma and astrocytoma.^81,143–145^ Even though the overexpression of enzymes involved in both the synthesis and breakdown of H_2_S has thus far only been reported for colorectal cancer,^134^ it can be envisaged that overexpression of both enzymatic pathways might be a common trait for any cancer type where H_2_S production is increased, as excess H_2_S can become toxic even for the most robust cancer cells (see below).

The exact manner by which cysteine-derived enhanced H_2_S metabolism contributes to cancer development is still to be fully clarified, although some common trends can be established. Different lines of evidence show that H_2_S modulates several aspects related to cancer cell adaptation within the tumour microenvironment.

### H_2_S in cancer: metabolism and bioenergetics

Perhaps the most well-established role of H_2_S in cancer cells is its contribution to stimulating cell bioenergetics and glycolytic metabolism. At subtoxic concentrations, H_2_S has been shown to stimulate ATP production at the level of oxidative phosphorylation, both as a source of electron equivalents for the mETC (via SQR-mediated quinone reduction, Table 1), and through per-sulphidation of ATPase, which maintains the enzyme in its activated state.^146^ Szabo et al.^52^ reported that increased oxygen consumption by mitochondria isolated from colorectal cancer HCT116 cells treated with cysteine was suppressed upon CBS inhibition. Similarly, in ovarian cancer cells, CBS inhibition resulted in mitochondrial dysfunction and ROS overproduction (Table 1), consistent with a malfunctioning mETC.^58^ Mitochondrial bioenergetics are also stimulated by the H_2_S-generating 3-MST substrate 3-MP (Table 1) both in the murine colon cancer CT26 cell line^147^ and in mouse hepatoma cells,^148^ where silencing of 3-MST or SQR decreased basal cellular bioenergetics, further suggesting that mitochondrial bioenergetics are partially sustained by SQR-mediated H_2_S oxidation (Table 1). In line with these observations, exposure of the SW480 colorectal cancer cell line to *n*-acetylcysteine yielded a synchronous upregulation of 3-MST and SQR expression and activity.^114^

In contrast to the stimulation of ATP production by low H_2_S levels, higher H_2_S concentrations inhibit complex IV of the mETC and thereby impair ATP production. Libiad et al.^134^ established a link between the increased expression, and changes in the localisation, of SOP enzymes and suppression of the growth-restricting effects of excess H_2_S in colorectal carcinoma cells. Indeed, given that colorectal cancer cell lines display increased expression of H_2_S-synthesising enzymes, increased expression of SOP enzymes affords a higher capacity of these cells to dispose of excess H_2_S while stimulating cell bioenergetics.

Stimulating effects of H_2_S on glycolytic metabolism in cancer cells have been demonstrated to result from per-sulphidation of lactate dehydrogenase (LDH, particularly the LDH-A isoform)^149,150^ and glyceraldehyde-3-phosphate dehydrogenase, although the functional consequences of per-sulphidation of the latter appear to be controversial.^151,152^

### H_2_S and cancer: beyond disturbed metabolism

In addition to the energetic stimulus afforded by low H_2_S concentrations, the link between the adaptability of cancer cells to an evolving and challenging environment and enhanced H_2_S metabolism extends to adaptation to hypoxia, antioxidant capacity, neoangiogenesis, cell cycle regulation, apoptosis evasion and chemoresistance. CBS and CSE have been reported to re-localise to mitochondria in response to hypoxia, a common feature of the tumour microenvironment, resulting in targeted H_2_S delivery that can stimulate ATP production and protect mitochondria from oxidative stress.^109,153^ Accordingly, exposure of SW480 colorectal cancer cells to hypoxia resulted in an enrichment in mitochondria of SQR protein levels and H_2_S detoxification activity.^154^ Besides the bioenergetics stimulation, the antioxidant nature of H_2_S is expected to protect cancer cells from oxidative damage. Indeed, protein per-sulphidation is posited to confer a protection mechanism to prevent irreversible oxidation of protein cysteine residues.^155^

The dysregulated proliferation of cancer cells leads to nutrient and oxygen deprivation, underlying the need for the formation of new blood vessels. The roles of CBS and CSE in neoangiogenesis have been shown for different cancer types, and possibly involve per-sulphidation of KATP channels and the activation of MAPK signalling pathways,^156–160^ which have been demonstrated in endothelial cells. Several studies have shown that H_2_S itself activates angiogenesis in cancer,^161–164^ evidence that was used to develop strategies to promote the release of H_2_S and the activation of angiogenesis under certain pathological circumstances, such as wound healing^165,166^ and inflammatory diseases.^167^

Evasion of apoptosis brought about by H_2_S has been attributed mostly to CSE-generated H_2_S mediating the per-sulphidation of key proteins in different signalling pathways: nuclear factor-κB (NF-κB) in hepatoma cells,^168^ the Keap1-transcriptional regulator of NRF2,^169^ and the extracellular-signal-regulated kinase (ERK)-activating protein kinase 1. CBS has been implicated in colorectal cancer carcinogenesis, its overexpression being detected even in precancerous lesions, such as hyperplastic polyps. Therefore, despite being only a partial effector of carcinogenesis, CBS belongs to a panel of intervening players, including NF-κB, KRAS, p53 and Wnt components, that contribute to metabolic rewiring and increased proliferative and invasive potential.^170^ Emerging evidence implies a role for CBS in the resistance of cancer cells to ferroptosis,^85,171^ although the mechanistic details of this observation remain to be unravelled (detailed below).

## Cysteine plasma pools and bioavailability

In plasma, cysteine is the major thiol that contributes to glutathione levels and protein synthesis.^172^ Under normal conditions, protein synthesis prevails over the other cysteine-dependent pathways. Although the degradation of glutathione contributes to the cysteine pool, the resulting levels are not sufficient for ‘normal’ metabolism upon cystine scarcity.^173^

**Fig. 4:**
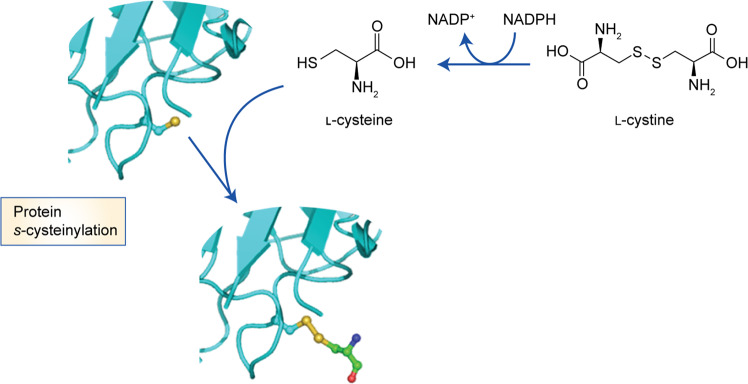
Protein *s*-cysteinylation. The oxidation of a cysteine residue within a protein can result in the formation of a cysteinyl radical. l-Cystine is reduced to l-cysteine under the action of l-cystine reductase. Reaction between protein cysteinyl residues and low molecular weight thiols such as free cysteine can yield *s*-cysteinylated proteins.

In healthy volunteers, the total cysteine availability in plasma is 200–300 μM, distributed across three pools—free reduced, free oxidised and protein bound. Up to 65% of cysteine is bound to proteins (protein *s*-cysteinylation; Fig. 4)^174,175^ and this pool increases with age.^176^ The remaining cysteine circulates mostly as cystine (25–30%, 40–50 μM) and the low abundance pool constituted by reduced cysteine. The concentration of cystine in blood is higher in women than in men and also increases with age.^177^ Cystine bioavailability across various tissues is ensured by different strategies, including drug-transporter-dependent mechanisms. The ability of NRF2 to regulate xCT coupled with the decline of NRF2 with age^178^ might account for the increased levels of plasma cystine seen with increasing age. Plasma from xCT-knockout mice contains a higher proportion of oxidised cysteine^179^ and xCT expression is increased in many tumours,^180^ pointing out its relevance in the context of cancer and eventually contributing for cancer metabolic rewiring. As many cysteine-containing proteins (transporters, receptors and enzymes) at extracellular surfaces or in extracellular fluids are prone to oxidation, their activity might be influenced by the thiol/disulphide redox microenvironment.^181^

## Total plasma cysteine as a marker of cancer risk

The level of total plasma cysteine (free reduced, free oxidised and protein bound) has been associated with the risk of developing cancer. For instance, increased cysteine availability in plasma is related to an enhanced risk of breast cancer, particularly oestrogen receptor-positive (ER^+^) and/or progesterone receptor-positive (PR^+^) cancer and in combination with low folate availability in plasma.^182^ In breast cancer, higher cysteine plasma concentrations are found in patients with higher body weight,^182,183^ which is a known risk factor for breast cancer, progression and mortality,^184^ further linking cysteine, metabolic dysregulation and cancer. However, a correlation between higher plasma cysteine levels and increased breast cancer risk was not observed in another prospective nested case–control study, wherein an inverse cysteine–cancer risk relationship was particularly evident in leaner women^183^ or related to the catechol-O-methyltransferase (COMT) genotype compatible with high enzymatic activity.^185^ Inconsistencies in the outcome of these studies might be attributed to the heterogeneity of the populations studied and/or different conditions of analysis and concentrations of plasma cysteine used. On the other hand, the association with the COMT genotype^185^ and ER^+^ breast cancer^182^ places cysteine metabolism as a relevant feature of oestrogen-dependent hormonal breast cancer. COMT o-methylates catechol oestrogen metabolites and mediates their detoxification, and it is plausible that the activity of this enzyme is inhibited by the accumulation of the cysteine precursor homocysteine.^185^ These toxic oestrogen metabolites are also detoxified by glutathione, which is therefore dependent on cysteine availability. Conjugates formed between glutathione and toxic metabolites are further metabolised into cysteinyl-s-conjugates, which have long half-lives in circulation.^186^ In fact, increased levels of cysteinyl-s-conjugates in biological fluids have been associated with several cancers, including melanoma, non-Hodgkin lymphoma, breast, ovarian and thyroid carcinomas, and with poor prognosis, recurrence and survival (see below).^186^

On the other hand, plasma cysteine levels have been reported to show an inverse correlation with the risk of cervical dysplasia, showing a weak negative association between the levels of cysteine and the development of low-grade, but not high-grade, squamous intraepithelial lesions in a large case–control study.^187^ Similarly, higher serum concentrations of cysteine were associated with a significantly reduced risk of oesophageal squamous cell carcinomas and gastric adenocarcinomas.^188^ In another study conducted in male smokers, high serum cysteine levels were associated with gastric adenocarcinomas but not with oesophageal squamous cell carcinomas^189^ or head and neck squamous cell carcinoma.^189^ The plasma cysteine concentration was inversely related to the incidence of colorectal cancer in postmenopausal women, for rectal and proximal tumours (*P* = 0.06), but not for distal tumours.^190^ Furthermore, the association was significant for localised tumours, but not for metastases, and was not observed in a study conducted only in men^191^ or other cohorts of non-postmenopausal women or women at different physiological stages.^192,193^ In a metabolomics study, the levels of cysteine were inversely related to overall glioma risk, being lower in the circulation of glioma patients compared with controls years in advance of diagnosis.^194^ This result was consistent with the accumulation of CSA in patient-derived low-grade glioma and with the intra-tumoural expression of CDO1,^76^ since the decreased level of cysteine can be due to the overexpression and activity of CDO1 found in high-grade glioblastomas.

Interestingly, higher plasma levels of cystine indicated a high probability of response in patients with non-small cell lung cancer before and after treatment with the immune checkpoint inhibitor nivolumab, which targets programmed cell death protein 1.^195^ Considering everything stated before implying cysteine as relevant to sustain the high performance (survival and proliferation) of cancer cells, acting as a detoxifying component or as an energy or biomass source; this observation can be a clue to use cysteine levels to predict the therapy response and the behaviour of tumours.

## Cysteine fractions and protein *s*-cysteinylation

Sullivan et al.^196^ showed that cystine levels are lower in tumour interstitial fluid in murine pancreatic adenocarcinomas (PDACs) than in plasma, which may indicate that cancer cells actively uptake cysteine. In the same work, cystine availability was lower in autochthonous PDAC tumours compared with subcutaneous tumours, supporting the relevance of anatomical location for the metabolic microenvironment. Nunes et al.^197^ reported increased total cysteine availability in the serum of women with ovarian tumours, regardless of whether the tumours were benign or malignant. The distinction between malignant and benign phenotypes was established by the presence of lower levels of free cysteine in the plasma of women with benign tumours. In addition, protein *s*-cysteinylation levels distinguish healthy controls from those with neoplasms, suggesting that discriminating plasma cysteine pools might be valuable for early diagnosis and outcome prediction. Patients ascites fluid, which is representative of the ovarian tumour microenvironment, was also rich in cysteine, derived predominantly from the s-cysteinylated form of albumin in plasma.^176^ S-cysteinylated proteins were found to be abundant in the ascites fluid and plasma of patients with ovarian cancer.^197^

### Micropinocytosis as an entry mode for cysteine and cysteine

Cysteine, in addition to other amino acids, is also made available to tumour cells though micropinocytosis of extracellular proteins.^198^ Several pathways have been implicated in micropinocytosis events, such as the ERK/MAPK pathway activated by oncogenic RAS, and PI3K/mTOR signalling pathways.^199^ As previously mentioned, due to its reactivity, cysteine is not an abundant core residue in protein sequences. However, in plasma most proteins are reversibly post-translationally modified by cysteinylation^114^ and, as mentioned above, s-cysteinylated albumin represents a major source of cysteine for cells, including cancer cells.^13^ Although the cellular pathways required for protein *s*-cysteinylation are not fully elucidated (Fig. 4), evidence shows that CBS might have a relevant role in this setting, as no s-cysteinylated albumin was detected in CBS-deficient mice.^200^

Disulphide-containing proteins have long been reported to be the major source of cystine in lysosomes via endoproteolysis.^201^ This evidence reinforces the role of micropinocytosis as a route for the import of different compounds (proteins), to be used as nutrients or signalling molecules in cancer promotion.^202,203^ Essentially, the imported disulphide-containing proteins undergo cathepsin-catalysed degradation in the lysosome, leading to the formation of cystine, which is then effluxed by the cystinosin transporter into the cytosol, where it is reduced to cysteine in a process that requires NADPH, leading to the formation of oxidised glutathione.^204,205^ Both thiol availability and redox status have been shown to influence the expression of cystinosin, and a shift towards a more oxidised status of cysteine and glutathione with a progressive increase in the mRNA levels of cystinosin has been reported. So, cysteinylated proteins taken up from the plasma by micropinocytosis can constitute a source of cysteine that once in the cell will be used in the different cysteine metabolic pathways, even in the *s*-cysteinylation of other proteins.

### Protein *s*-cysteinylation in cancer

Although the role of protein *s*-cysteinylation has not been fully explored in humans, some insights into its role in cancer, relating to the *s*-cysteinylation of certain proteins with cancer features, have emerged. For example, heparinase, an enzyme that degrades heparan sulphate and enhances the invasive and metastatic capacity of cancer cells, was reported to be activated by *s*-cysteinylation.^206^ In addition, enzymes involved in the control of oxidative stress can be modulated by *s*-cysteinylation, as is the case for the metalloenzyme Cu/Zn-superoxide dismutase 1 (SOD1), which catalyses the dismutation of superoxide anions into oxygen and hydrogen peroxide. *s*-Cysteinylation of SOD1 prevents the inhibitory action of hydrogen peroxide on SOD1,^207,208^ which contributes to an increased antioxidant potential of cancer cells and thereby protects them from oxidative damage. In fact, the reactive species interactome has been the subject of a very interesting review stating that the adaptive, selective and evolutionary cellular mechanisms that are required to overcome oxidative stress are largely dependent on cysteine redox switches, leading to enzyme modification and improved antioxidant capacity.^209^ Furthermore, *s*-cysteinylation of proteins and peptides from major histocompatibility complex classes I and II has been outlined as a means of reducing the immune effectiveness of T-lymphocytes,^210^ which is important in cancer as it can promote immune evasion and reduce the elimination of cancer cells by the immune system.

## Therapeutic strategies targeting cysteine metabolism in cancer

**Fig. 5:**
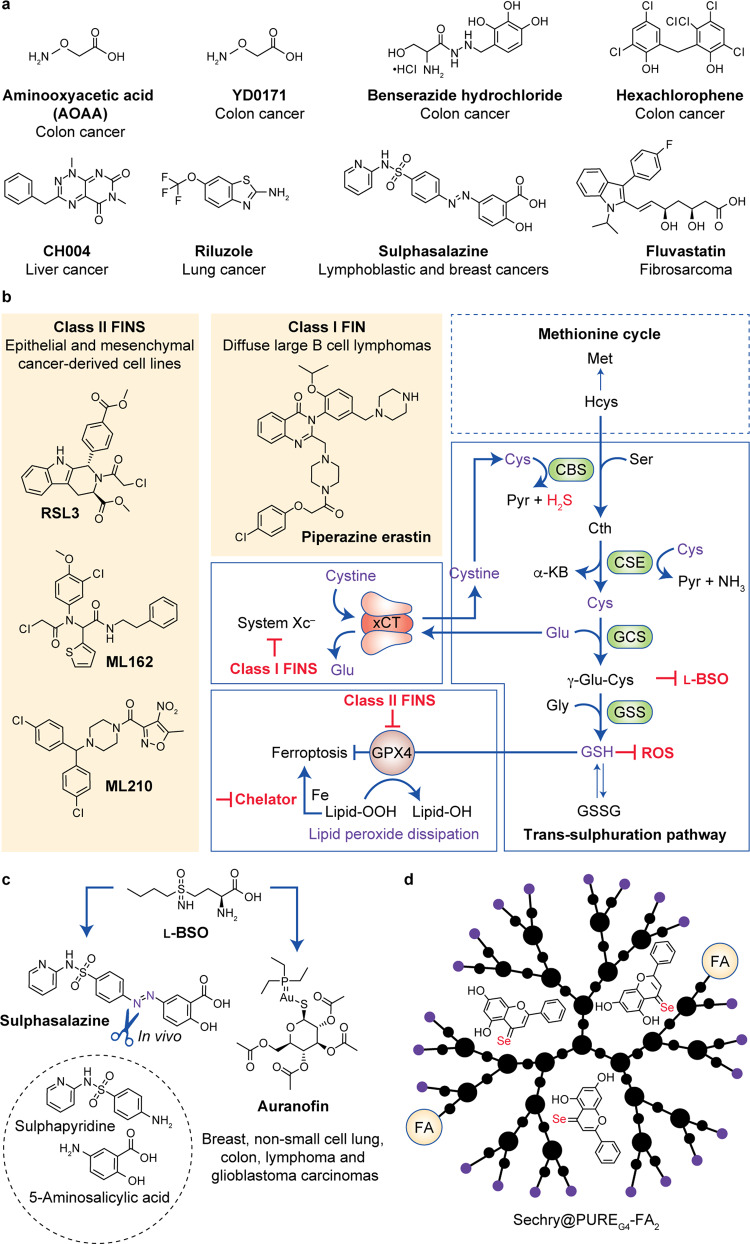
Targeting cysteine metabolism. **a** Drugs targeting cysteine metabolism. **b** Key inhibition targets and interplay between the trans-sulphuration pathway, the xCT antiporter and ferroptosis. **c** Inhibition of glutathione- and thioredoxin-dependent antioxidant pathways by sulphasalazine or auranofin combined with L-BSO. **d** Novel selenium-chrysin nanoformulations acting as inhibitors of glutathione bioavailability/synthesis and CBS activity. CBS cystathionine β-synthase, CSE cystathionine γ-lyase, GCS γ-glutamyl cysteine synthetase, GSS glutathione synthetase, Pyr pyruvate, α-KB α-ketobutyrate, GSH glutathione reduced form, GSSG glutathione oxidised form, ROS reactive oxygen species, FIN ferroptosis-inducing compounds.

Throughout this review, the trans-sulphuration pathway has been presented as a metabolic pathway that involves the interconversion (and de novo synthesis) of cysteine and homocysteine through the intermediate cystathionine, cysteine being a critical component of glutathione (Fig. 5b),^211^ and both cysteine and homocysteine contributing to the production of H_2_S. As stated before, glutathione is the most abundant antioxidant in the cell, with a key role in ROS scavenging in cancer cells.^212^ However, clinical trials aimed at inhibiting glutathione synthesis were unsuccessful, despite some strategies having provided promising results.^213^ Instead, inhibition of the H_2_S-synthesising trans-sulphuration pathway enzymes CBS and CSE, together with 3-MST, still holds great promise for cancer treatment,^81,143,214^ as outlined below. Another strategy outlined below relies on inhibition of the xCT system, which mediates the entry of cystine that fuels the trans-sulphuration pathway. Other therapeutic approaches outlined below include systemically decreasing cysteine levels and inhibiting GPX4.

### Systemic depletion of cysteine

Strategies to bring about the systemic decrease of cysteine levels are advanced in the management of cancer, and some studies have presented promising results in breast, prostate and leukaemia models in vivo. These studies report a significant reduction in tumour burden upon the systemic depletion of cysteine using cyst(e)inase.^215^ Cyst(e)inase degrades extracellular cysteine and cystine and leads to decreased levels of intracellular cysteine and glutathione, which profoundly affects the redox potential of cancer cells^216^ and counteracts the essential role of cysteine in cancer cells.^80^ Cystine starvation has been reported to play an important role in cell death in different types of cancer. The mechanism is not fully understood but it is believed that the increased intracellular glutathione levels and increased ROS^217^ are closely coupled with the build-up of toxic lipid peroxides and consequent ferroptosis.^218,219^

### Targeting H_2_S biosynthesis

The inhibition of H_2_S biosynthesis has been shown to sensitise cancer cells to chemotherapy, as it disturbs the mitochondrial equilibrium by impairing bioenergetics.^220–222^ Thus, the blockade of enzymes that contribute to the production of H_2_S constitutes an attractive anti-cancer strategy. As such, drug screening targeting CBS, CSE or 3-MST has been conducted; however, as yet, no inhibitor with pharmacological potential has been identified.^114,220,223–225^ Different CBS inhibitors have been designed and tested over the past years, starting with amino-oxyacetic acid (AOAA)^52,58,60^ and related prodrugs;^226^ unfortunately, however, AOAA was shown to also inhibit CSE activity.^227^ Subsequently, several compounds (e. g. hexachlorophene and benserazide) that were already clinically available were tested and repurposed as CBS inhibitors (with the knowledge that they were not specific for CBS).^225^ In 2018, Wang et al.^171^ designed and synthesised a novel, potent and bioactive inhibitor of CBS. This pharmacological probe (CH004, Fig. 5a) allows selective inhibition of CBS (raising cellular homocysteine levels and suppressing the production of H_2_S in a dose-dependent manner) over CSE; it can suppress cell proliferation with cell cycle arrest at S phase and, notably, it can reduce tumour growth in a xenograft mouse model. Importantly, the underlying cell death mechanism is triggered by CBS inhibition in HepG2 cells via ferroptosis, suggesting that CBS has a previously unreported function in this process. Nevertheless, further studies are required to elucidate the exact role and underlying molecular mechanisms of the trans-sulphuration pathway in cancer cell death.

In contrast to inhibiting H_2_S biosynthesis, some studies have addressed the advantage of ‘poisoning’ cancer cells with high levels of H_2_S, by using donors that release H_2_S in the tumour and somehow block the most important signalling pathways sustaining cancer survival, such as PI3K and MAPK.^228–230^

### Inhibiting xCT

The search for strategies and drugs aimed at inducing oxidative stress has revealed xCT to be a suitable target—increased glutamate efflux through this antiporter is an important process for cells to generate sufficient glutathione to cope with high intracellular ROS levels.

Wangpaichitr et al.^231^ found that riluzole (Fig. 5a), approved for the treatment of amyotrophic lateral sclerosis, increases ROS levels by multiple mechanisms, including decreasing LDH-A and NAD^+^ levels to increase oxidative stress, as well as interfering with the xCT antiporter to block the cystine–glutamate pump. Together, these two mechanisms seem to work jointly to enhance ROS levels and lead to cell death in cisplatin-resistant lung cancer cells; this is an important achievement as no drugs are available to overcome cisplatin resistance or kill cisplatin-resistant cells.

Sulphasalazine (Fig. 5a), a pro-drug that combines sulphapyridine (an antibiotic) and 5-aminosalicylic (an anti-inflammatory agent) linked by an azo bridge, has huge therapeutic potential, but is, unfortunately, labile under physiological conditions (70% degradation by colonic bacteria via azo cleavage; Fig. 5c). Nevertheless, sulphasalazine has been demonstrated to be an effective treatment in cancer models, by inhibiting xCT to activate ferroptosis^232,233^ and restore sensitivity to chemotherapy.^234^ Gout et al.^235^ also reported that targeting xCT with sulphasalazine potently suppresses lymphoma growth. The effect of sulphasalazine on reducing the ROS defence capacity of cancer cells and sensitising them to available chemotherapeutic drugs (e.g. cisplatin, docetaxel) is associated with the activation of p38 MAPK-mediated growth suppression.^236^

As well as triggering cell death, xCT inhibition also induces the metabolic rewiring of cancer cells. Timmerman et al.^237^ detected metabolic responses related to perturbations in glutamine metabolism in 47 independent breast cancer-derived cell lines, meaning that the inhibition of xCT promotes an adjustment in glutamine metabolism, which indirectly contributes to the import of cysteine through the exchange with glutamine-derived glutamate through xCT. This metabolic adaptation can be relevant in triple-negative breast cancers (those lacking ERs, PRs and HER2, which constitute approximately a quarter of breast tumours) that express xCT, which exhibit increased levels of ROS induced by decreased glutamate levels upon glutamine scarcity. The authors hypothesised that xCT inhibition might be further potentiated by limiting glutamate or glutamine availability, and show that xCT is a compelling therapeutic target for triple-negative tumours. Okazaki et al.^238^ studied genes related to glutaminolysis in order to determine the sensitivity of glutamine metabolism to xCT-targeted therapy in head and neck squamous cell carcinoma. A metabolome analysis disclosed that sulphasalazine triggers an increase in the glutamate-derived TCA cycle intermediate α-ketoglutarate in addition to a decrease in cysteine and glutathione. This observation means that xCT blockage induces the accumulation of glutamate that is converted into α-ketoglutarate instead of being used in glutamate-cysteine exchange and consequently that is why cysteine does not enter the cell and give rise to glutathione.

### GPX4 inhibition

Through the metabolomic profiling of 177 cancer cell lines, Yang et al.^239^ showed that glutathione depletion constitutes one mechanism of ferroptosis. Two classes of ferroptosis-inducing compounds were investigated (Fig. 5b), based on different approaches for inhibiting GPX4 (Fig. 4b). One of these classes (class I ferroptosis-inducing compounds, erastin derivatives) inhibits GPX4 through the depletion of glutathione, and the other (class II ferroptosis-inducing compounds, RSL3 derivatives) inhibits GPX4 directly, without glutathione depletion. Indirect inhibition of GPX4 using l-buthionine sulphoximine (L-BSO; an inhibitor of GCL and consequently of glutathione synthesis) enhanced ferroptotic cell death induced by all ferroptosis-inducing compounds, and its modulation effect is specific to ferroptosis-inducing compounds. Later, Viswanathan et al.^240^ identified ML210 and ML162 as two new class II ferroptosis-inducing compounds. In this study, it was also demonstrated that treatment with the inhibitor of cholesterol synthesis fluvastatin (Fig. 5a) led to a decrease in the expression of GPX4 in a time- and concentration-dependent manner, and a cumulative effect was observed when using fluvastatin and RSL3, showing that both contribute to ferroptosis.

### Drug combinations

Combinations of agents that target cysteine metabolism have also been explored. Harris et al.^5^ combined L-BSO with sulphasalazine or auranofin (a gold salt used in the treatment of rheumatoid arthritis) to inhibit both glutathione- and thioredoxin-dependent antioxidant pathways, triggering synergistic cancer cell death (Fig. 5c). Ye et al.^241^ investigated the effect of the combined administration of paclitaxel and RSL3: alone and at low concentrations, these agents do not cause substantial cell death. Low-concentration paclitaxel (3–6 nM) is reported to interfere with glutaminolysis, a process that is essential for ferroptosis. Remarkably, the combination of these drugs induced ferroptosis and significant cell death in p53-mutated hypopharyngeal squamous cell carcinoma. Also, low-concentration paclitaxel (2 nM) enhanced RSL3-induced ferroptosis by upregulating the expression of p53 variants

### Nanoformulations

Nanoformulation using targeted nanoparticles such as dendrimers is an emerging strategy in cancer therapeutics. Precise drug delivery by dendrimer nanoparticles is easily achieved by surface targeting, and folate-targeted dendrimers in particular are a relevant choice in cancer therapeutics, because cancer cells overexpress the folate receptor.^242^ Mota et al.^243^ developed folate-targeted dendrimers that are able to load and release L-BSO, whereas Santos et al.^244^ also used nanoparticles to produce selenium-chrysin (SeChry) nanoformulations to target glutathione bioavailability and CBS, aiming at novel ovarian cancer therapeutics (Fig. 5d). SeChry was chosen as a plausible competitive inhibitor of xCT as xCT is also able to take up selenium. Interestingly, this nanoformulation increased the specificity for SeChry delivery to ovarian cancer cells, as the nanoparticles were functionalised with folate and cancer cells express more folate receptor, and therefore significantly reduced the toxicity against non-malignant cells. Although SeChry did not affect the uptake of cysteine, it did increase glutathione depletion, indicating that it might induce oxidative stress, which will be scavenged by GPX4, a selenium-dependent enzyme.^245^ Also, in vitro enzymatic assays revealed an inhibitory effect of SeChry towards CBS, thus inhibiting H_2_S production.^244^

## Conclusions and future perspectives

Once inside a cell, cysteine can have different fates, including the synthesis of glutathione or proteins, oxidative or non-oxidative catabolism, and reversible post-translational protein modification, each of which can contribute to the development and progression of cancer (Fig. 1). Having such a wide relevance in cancer, cysteine metabolism is undoubtedly a relevant target.

As mentioned above, a number of compounds or drug formulations have been investigated with regard to inhibiting cysteine metabolism, either by targeting H_2_S-synthesising trans-sulphuration pathway enzymes (e.g. CBS) or via xCT inhibition. However, there are many more studies that need to be carried out, and the description of ferroptosis—a relatively unexplored cancer cell death mechanism that is inhibited by glutathione-dependent GPX4—reinforces the importance of cysteine in cancer and points to new perspectives regarding the ways whereby cysteine import and synthesis and glutathione synthesis can be efficiently targeted in order to trigger cell death in cancer. Targeted nanoformulation is a very efficient and attractive platform that might solve the issues of low solubility and metabolic stability of some therapeutic agents. Selenium-containing drugs are expected to lead to important advances in novel cancer therapeutics, due the involvement of GPX4 in the elimination of lipid peroxides and in ferroptosis prevention. As well as chemical targeting, enzymatic targeting is another challenging strategy and cyst(e)inase-mediated depletion of serum cysteine and cystine pools has already been demonstrated to suppress the growth of tumours in different animal models.^215^

Herein, we describe the multiple possibilities whereby an enhanced cysteine transport and metabolism enables various types of cancer cells to adapt to the challenging tumour microenvironment, and to thrive, proliferate and acquire chemoresistance. This cysteine-centred view of cancer biology offers various possibilities to develop new and improved diagnostics tools and pharmacological strategies to target cancer.

## Data Availability

All data presented in the review paper is published and referenced.
